# Chromophobe Renal Cell Carcinoma With Sarcomatoid Differentiation: A Case Report and Review of the Literature

**DOI:** 10.7759/cureus.83143

**Published:** 2025-04-28

**Authors:** Mouhsine Omari, Mohammed Bendimya, Fadoua Jebrouni, Nassira Karich, Ouissam Al Jarroudi, Sami Aziz Brahmi, Amal Bennani, Said Afqir

**Affiliations:** 1 Medical Oncology, Mohammed VI University Hospital, Oujda, MAR; 2 Medical Oncology, Faculty of Medicine and Pharmacy, Mohammed First University, Oujda, MAR; 3 Anatomopathology, Faculty of Medicine and Pharmacy, Mohammed First University, Oujda, MAR

**Keywords:** chromophobe carcinoma, immune checkpoint inhibitors, prognosis, sarcomatoid differentiation, target therapy

## Abstract

Chromophobe renal cell carcinoma is a rare entity with an excellent prognosis compared with clear renal cell carcinoma and is characterized by distinct molecular and genetic specificity. The presence of a sarcomatoid component is an uncommon phenomenon, which indicates a high risk of metastasis and a poor prognosis. We present the case of a 44-year-old patient with chromophobe renal cell carcinoma with a sarcomatoid component. Therapeutic management presents a significant challenge given the absence of standards of care for this rare entity. The current treatments are based on vascular endothelial growth factor tyrosine kinase inhibitors, mammalian target of rapamycin pathway inhibitors, and immune checkpoint inhibitors. Close monitoring based on clinical, biological, and radiological examinations is necessary for rapid and appropriate interventions. Moreover, this histological variant represents a major clinical challenge, not only because of its aggressive behavior but also due to the absence of specific clinical manifestations and its frequent incidental discovery at an advanced stage, further complicating early diagnosis and management.

## Introduction

Chromophobe carcinoma represents the third histological subtype of kidney cancer after clear renal cell carcinoma and papillary carcinoma [1]. It accounts for 5% of all kidney cancers [1] and is distinguished by distinct molecular and genetic specificity. It is asymptomatic and usually discovered incidentally on imaging. The presence of symptoms is suggestive of advanced disease, and the prognosis for chromophobe carcinoma is excellent, with a survival rate of up to 80% to 90% [2].

However, the presence of sarcomatoid differentiation is a rare histological transformation that contains both carcinoma-like and sarcomatoid features. Its incidence is estimated to be between 2% and 11% [2]. Its presence indicates a poor prognosis with a high risk of metastasis [2].

The treatment of metastatic chromophobe renal cell carcinoma with a sarcomatoid component is complex due to the lack of clearly established therapeutic protocols. It is based on the use of tyrosine kinase inhibitors targeting the vascular endothelial growth factor, such as sunitinib, mammalian target of rapamycin (mTOR) pathway inhibitors like everolimus, and immune checkpoint inhibitors, notably pembrolizumab or nivolumab.

## Case presentation

**Figure 1:**
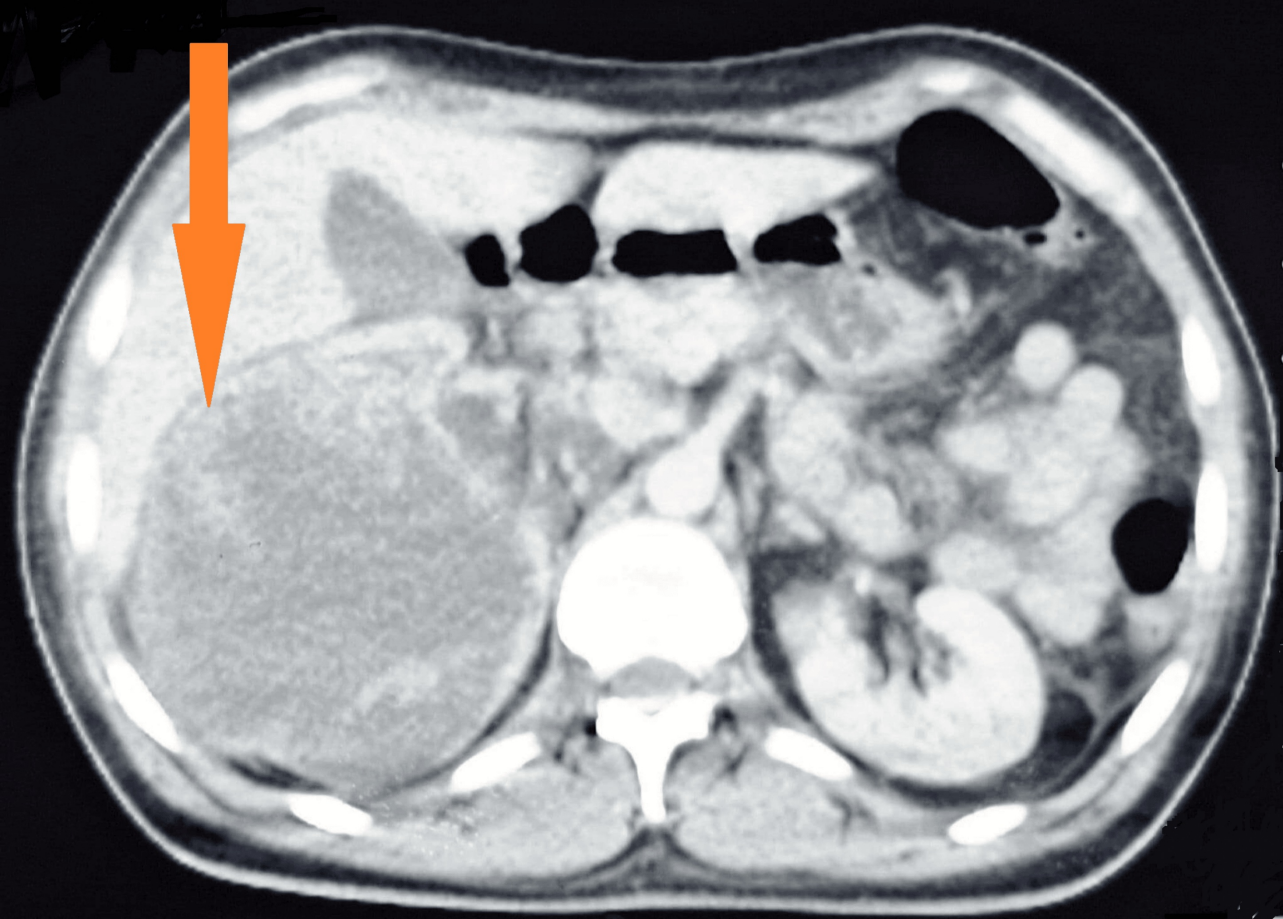
Axial CT scan image (arrow). The CT scan image shows a large right renal mass (arrow) with massive fluid containing thick partitions and a tissue portion.

We present the case of a 44-year-old patient with a history of type 2 diabetes managed with insulin, who was a chronic smoker (40 pack-year) with no history of drug use or allergy and no family history of similar cases. The disease onset occurred six months prior with right lower back pain, prompting the patient to seek outpatient treatment. A computed tomography (CT) scan revealed a voluminous right renal mass with massive fluid containing thick partitions and a tissue portion, moderate uretero-hydronephrosis with lithiasis of the inferior calcific group and an infracentimetric renal hilar (Figure 1). Subsequently, the patient was referred to the University Hospital for management.

The patient underwent an extensive work-up, which revealed a locally advanced necrotic right renal tumor process with endopyelic extension associated with infiltration of the surrounding fat, likely related to localized carcinosis, and retroperitoneal latero-aortic and inter-aorto-caval adenopathy. Bilateral pulmonary parenchymal micronodules were observed, to be considered in the context of the patient's condition. Mirror erosion and condensation of the upper C6 and lower C5 vertebral plateaus were noted. The work-up was supplemented by a cervical magnetic resonance imaging to investigate potential bone metastases, suggesting spinal cord conversion. A review of the imaging indicated the absence of metastases, particularly in the bone and lung.

**Figure 2:**
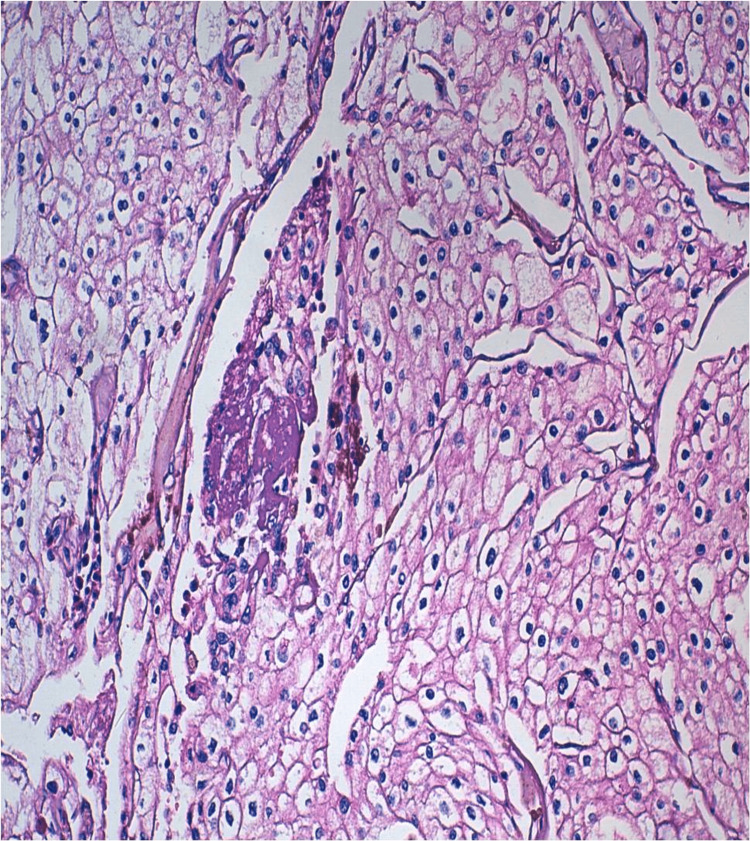
Microscopic findings of the right total nephrectomy specimen. Microscopic examination shows tumor proliferation made up of large cells with abundant acidophilic or eosinophilic cytoplasm with well-defined clear boundaries and an irregular, ovoid nucleus with coarse chromatin (hematoxylin and eosin, x200).

**Figure 3:**
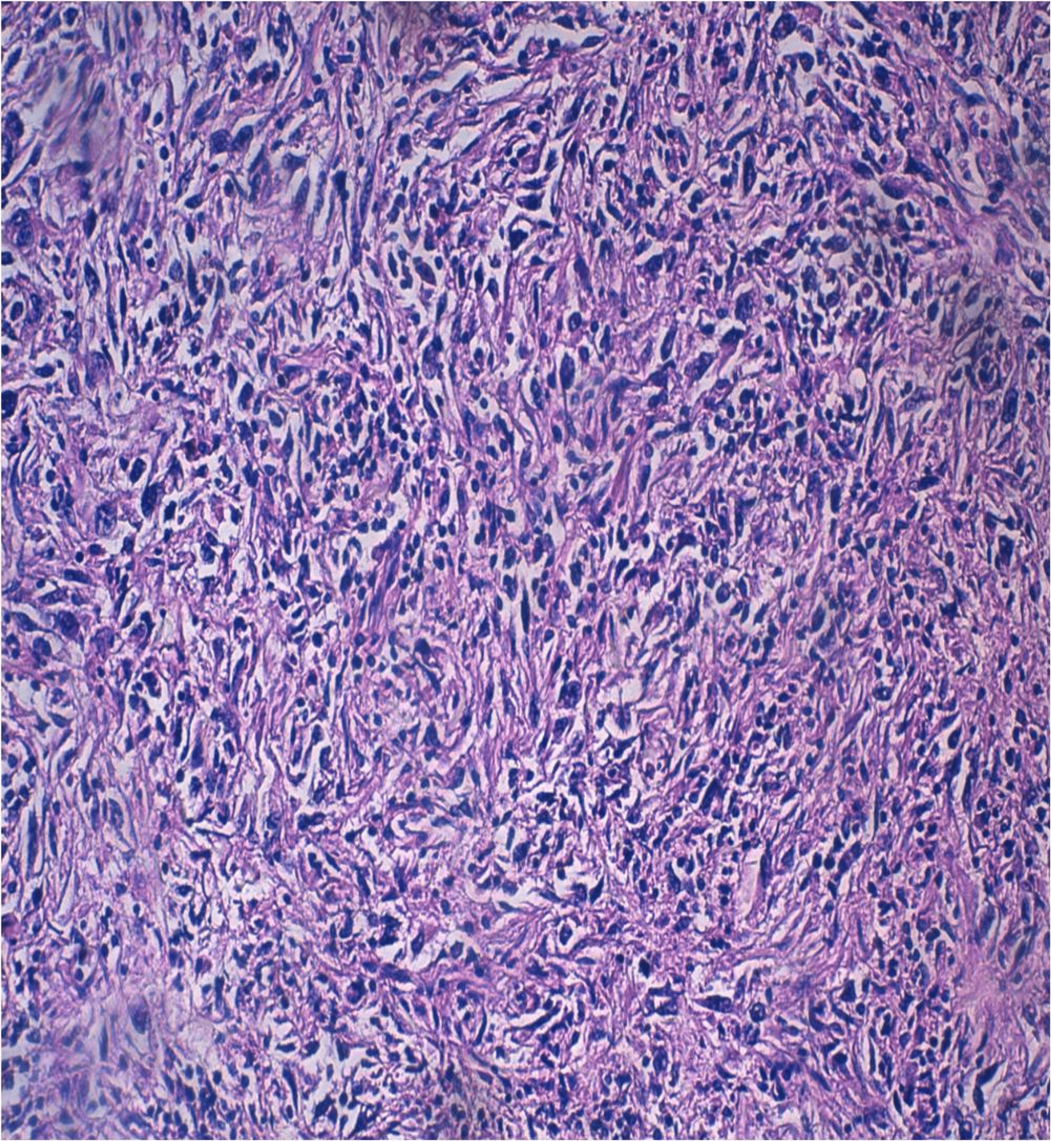
Microscopic findings of the right total nephrectomy specimen. Microscopic examination shows the presence of a sarcomatoid component (hematoxylin and eosin, x200).

The therapeutic decision made during the multidisciplinary consultation meeting was to perform a radical nephrectomy. The patient underwent an enlarged radical nephrectomy. Anatomopathological examination revealed a 12.5 cm-long tumor that had invaded the renal vein and pyelocaliceal cavities, exhibiting a macroscopic thrombus. The tumor was classified as pT3aN1Mx, with tumoral vascular surgical margins, positive lymph node dissection (two nodes positives out of three nodes), and extensive necrotic remodeling over 80% with a 40% sarcomatoid component and no rhabdoid component (Figures 2, 3).

**Figure 4:**
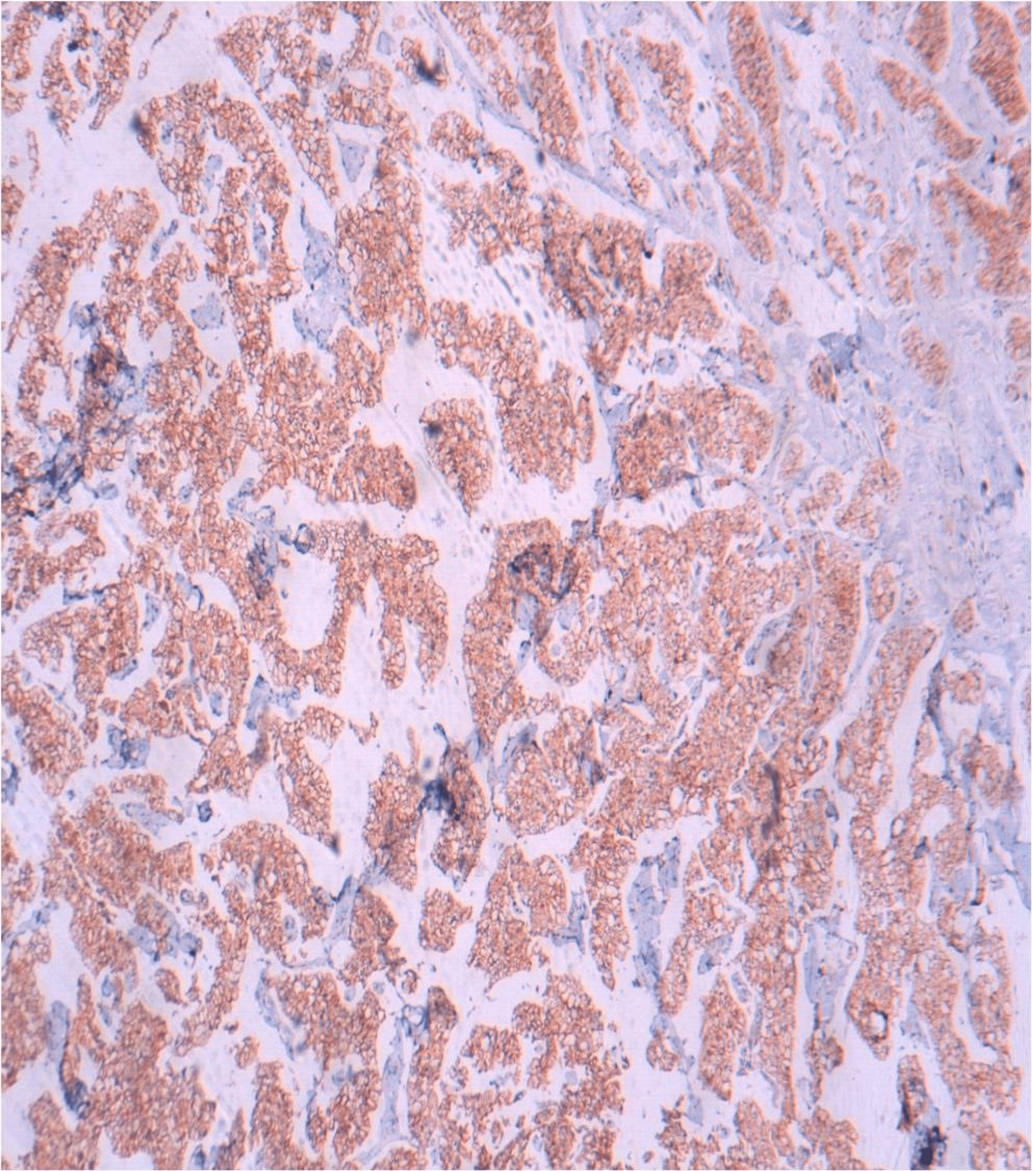
Immunohistochemical findings of the right total nephrectomy specimen. Immunohistochemical analysis shows diffuse membranous expression of CD117 (immunohistochemical staining, x200).

**Figure 5:**
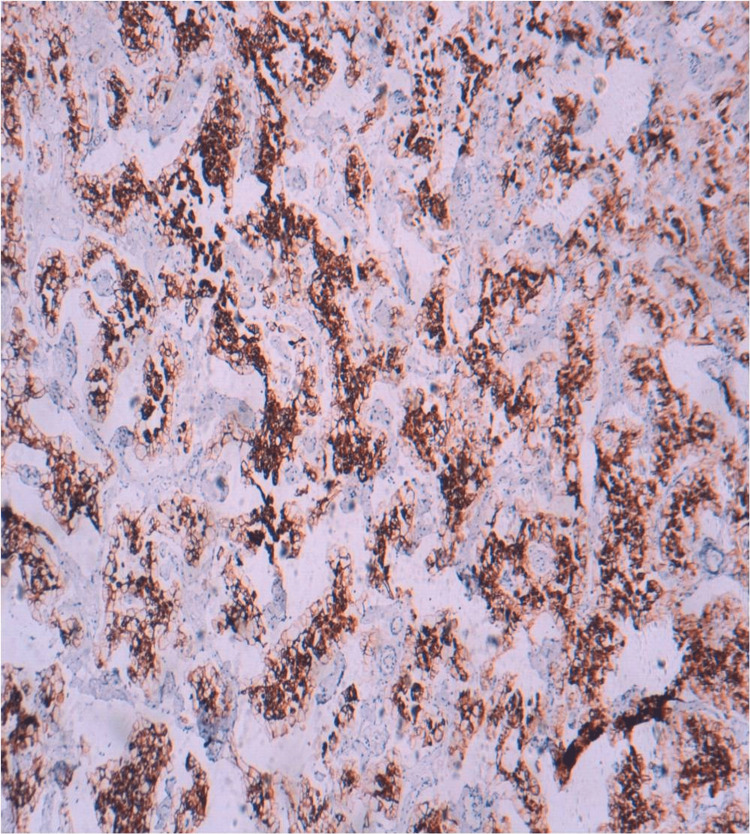
Immunohistochemical findings of the right total nephrectomy specimen. Immunohistochemical analysis shows diffuse cytoplasmic expression of CK7 (immunohistochemical staining, x200).

The immunohistochemical analysis revealed that the tumor cells were positively labeled with cluster of differentiation 117 protein (CD117) (Figure 4), cytokeratin 7 (CK7) (Figure 5), and epithelial membrane antigen and negatively labeled with vimentin and cluster of differentiation 10 protein (CD10).

The patient was referred to the oncology center for further treatment. The initial clinical examination revealed a conscious patient with hemodynamic and respiratory stability, a performance status index of 1, a clean nephrectomy scar, and free lymph nodes. The multidisciplinary team determined that a positron emission tomography (PET) scan was necessary to complete the diagnostic procedure. However, due to resource limitations and the high cost of the PET scan, the patient was placed under close surveillance.

After three months, the patient developed an inflammatory syndrome associated with intractable vomiting. Laboratory tests provided the following results, summarized in Table 1.

**Table 1:** Results of laboratory tests.

Laboratory test	Results	Reference Range
Leukocytes (cells per mm³)	17,610	4,000 - 10,000
Neutrophils (cells per mm³)	10,566	1,500 - 7,000
Eosinophils (cells per mm³)	3,170	100 - 400
C-reactive protein (mg/L)	177	<10
Procalcitonin (ng/L)	0.11	<0.5
Ferritin (ng/mL)	1,022	20 - 300
Amoebiasis (stool antigen test)	Positive	Negative

The patient was administered antiemetics and antibiotics, and a CT scan revealed secondary pleural and pulmonary locations, as well as diffuse tissue masses and nodules indicative of peritoneal carcinomatosis.

The multidisciplinary team decided to initiate treatment with sunitinib 50 mg/m², administered in cycles of four weeks ON and two weeks OFF, for three cycles before re-evaluating the patient. Despite initiation of targeted therapy, the patient's condition rapidly deteriorated, and he succumbed 15 days after starting treatment.

## Discussion

Chromophobe renal cell carcinoma is a rare anatomo-clinical entity initially described by Thoenes and Colls in 1985 [3] and constitutes 5% of all kidney cancers [1]. It originates in the intercalated cells of the collecting duct [4] and is categorized into three subtypes based on the proportion of cells: classic, eosinophilic, and mixed [4]. It is typically discovered incidentally through imaging, and the presence of an abdominal mass, hematuria, and abdominal pain is indicative of advanced tumors.

The identified risk factors for chromophobe renal cell carcinoma include elevated body mass index and tobacco use [5]. While predominantly sporadic, familial forms are infrequent and may manifest as genetic syndromes such as Birt-Hogg-Dubé syndrome [6] and Cowden syndrome (Phosphatase and Tensin Homolog (PTEN)) [7]. Molecular analysis is characterized by chromosomal aneuploidies, mutations in protein 53 (P53) and PTEN, and increased mitochondrial activity [8].

Chromophobe renal cell carcinoma presents a major diagnostic challenge due to the absence of symptoms observed at an early stage and the accidental discovery of the disease. In chromophobic renal cell carcinoma, Hale staining and the presence of intracytoplasmic microvesicles are characteristic features. Positive staining for CK7, CD117, and kidney-specific cadherin and negative staining for vimentin, carbonic anhydrase IX (CA9), and CD10 are essential markers in immunohistochemistry for confirming the diagnosis [9].

The presence of a sarcomatoid component is an uncommon finding in 9% of chromophobe renal cell carcinomas [10]. This component results from the process of epithelial-mesenchymal transformation and exhibits both carcinoma-like and sarcomatoid histological features, characterized by spindle cells, cellular atypia, and necrosis with microvascular invasion [10].

The prognosis is excellent, given that chromophobe carcinoma is most often restricted to the kidney and of low nuclear grade. Increased tumor size and sarcomatoid differentiation are poor prognostic factors and increase the risk of metastatic development [11]. Local and distant recurrence of chromophobe renal cell carcinoma is rare, accounting for 5-6% of cases [11]. The most frequent sites of metastasis are lymph nodes, lungs, bone, and liver [12].

Therapeutic management is a real challenge, given the lack of specific standards of care for this rare entity and the small number of patients enrolled in studies, as well as the fact that dedicated clinical data for chromophobe renal cell carcinoma are limited and excluded from phase 3 controlled trials or extrapolated from clear cell carcinoma algorithms. Our knowledge of molecular mechanisms enabled us to develop new therapeutic options, in particular anti-angiogenic agents, mTOR pathway inhibitors, and immune checkpoint inhibitors.

The preferred first-line treatment options are sunitinib and everolimus. Sunitinib, an oral multi-targeted inhibitor of vascular endothelial growth factor receptor tyrosine kinase, was evaluated in a single-arm prospective trial including 57 patients, only five of whom had chromophobe carcinoma. Progression-free survival was 12.7 months with an objective response rate of 40% [13]. Then the phase 2 trial (ASPEN) compared the activity of sunitinib 50mg/day for four weeks with a two-week rest to everolimus 10mg/day (mTOR pathway inhibitor) including 108 patients including 16 patients with chromophobe renal cancer and 16 patients with a sarcomatoid component. Progression-free survival was 11.4 months for everolimus versus 5.5 months for sunitinib [14]. A second prospective phase 2 trial (ESPN) included 68 patients including 12 patients with chromophobe renal cell carcinoma, progression-free survival was 8.9 months for sunitinib and not reached for everolimus, while overall survival was 31.6 months for sunitinib and 25.1 months for everolimus [15].

Cabozantinib is the preferred option according to National Comprehensive Cancer Network (NCCN) recommendations [16]. It was approved in a retrospective study including 30 patients of which six patients had chromophobe carcinoma and only one patient had a sarcomatoid component. Progression-free survival was 8.6 months, and overall survival was 25.4 months [17]. Another study was evaluated including 112 patients of whom 10 patients had chromophobe carcinoma and six patients had a sarcomatoid component, and the median time to treatment failure was 5.7 months for chromophobe carcinoma and 5.1 months for the presence of a sarcomatoid component with an overall survival at 12 months of 60% for chromophobe carcinoma alone and 25% for sarcomatoid differentiation [18].

Pazopanib 800 mg/day for four weeks was evaluated in a single-arm, open-label, multicenter, phase 2 trial involving 29 patients without sarcomatoid involvement and only three patients with chromophobe carcinoma. Progression-free survival was 18.3 months and overall survival was 18.9 months [19].

The activity of immune checkpoint inhibitors was confirmed by the phase 2 KEYNOTE-427 trial, which showed the efficacy of pembrolizumab 200mg/day every three weeks for 35 cycles. The objective response rate was 9.5% for chromophobe carcinoma [20]. Another retrospective study evaluated the clinical activity of nivolumab, including 41 patients, five of whom had chromophobe carcinoma. Stability was observed in three patients, progression in a single patient, and a patient who could not be evaluated. Progression-free survival for the study as a whole was 3.5 months [21].

The combination of a tyrosine kinase inhibitor (lenvatinib 18mg) with an inhibitor of the mTOR pathway (everolimus 5mg) was approved in a phase 2 multicenter trial including 31 patients, nine of whom had chromophobe carcinoma. Progression-free survival was 13.1 months with an objective response rate of 44% [22].

The available data on the medical treatment of patients with chromophobe renal cell carcinoma with a sarcomatoid component is highly limited; therefore, participation in specific clinical trials is strongly recommended.

## Conclusions

Metastatic chromophobe renal cell carcinoma is rare and presents a significant diagnostic and therapeutic challenge. The identification of sarcomatoid differentiation is essential because the chromophobe variant of renal cancer has a more favorable prognosis than the clear cell variant; however, the sarcomatoid component of chromophobe is paradoxically aggressive and associated with a poor prognosis.

## References

[1] Muglia VF, Prando A (2015). Renal cell carcinoma: histological classification and correlation with imaging findings. Radiol Bras.

[2] Ged Y, Chen YB, Knezevic A (2019). Metastatic chromophobe renal cell carcinoma: presence or absence of sarcomatoid differentiation determines clinical course and treatment outcomes. Clin Genitourin Cancer.

[3] Thoenes W, Störkel S, Rumpelt HJ (1985). Human chromophobe cell renal carcinoma. Virchows Arch B Cell Pathol Incl Mol Pathol.

[4] Störkel S, Steart PV, Drenckhahn D, Thoenes W (1989). The human chromophobe cell renal carcinoma: its probable relation to intercalated cells of the collecting duct. Virchows Arch B Cell Pathol Incl Mol Pathol.

[5] Purdue MP, Moore LE, Merino MJ (2013). An investigation of risk factors for renal cell carcinoma by histologic subtype in two case-control studies. Int J Cancer.

[6] Menko FH, van Steensel MA, Giraud S (2009). Birt-Hogg-Dubé syndrome: diagnosis and management. Lancet Oncol.

[7] Shuch B, Ricketts CJ, Vocke CD (2013). Germline PTEN mutation Cowden syndrome: an underappreciated form of hereditary kidney cancer. J Urol.

[8] Davis CF, Ricketts CJ, Wang M (2014). The somatic genomic landscape of chromophobe renal cell carcinoma. Cancer Cell.

[9] Liu L, Qian J, Singh H, Meiers I, Zhou X, Bostwick DG (2007). Immunohistochemical analysis of chromophobe renal cell carcinoma, renal oncocytoma, and clear cell carcinoma: an optimal and practical panel for differential diagnosis. Arch Pathol Lab Med.

[10] de Peralta-Venturina M, Moch H, Amin M (2001). Sarcomatoid differentiation in renal cell carcinoma: a study of 101 cases. Am J Surg Pathol.

[11] Moch H, Ohashi R (2021). Chromophobe renal cell carcinoma: current and controversial issues. Pathology.

[12] Motzer RJ, Bander NH, Nanus DM (1996). Renal-cell carcinoma. N Engl J Med.

[13] Tannir NM, Plimack E, Ng C (2012). A phase 2 trial of sunitinib in patients with advanced non-clear cell renal cell carcinoma. Eur Urol.

[14] Armstrong AJ, Halabi S, Eisen T (2016). Everolimus versus sunitinib for patients with metastatic non-clear cell renal cell carcinoma (ASPEN): a multicentre, open-label, randomised phase 2 trial. Lancet Oncol.

[15] Tannir NM, Jonasch E, Albiges L (2016). Everolimus versus sunitinib prospective evaluation in metastatic non-clear cell renal cell carcinoma (ESPN): a randomized multicenter phase 2 trial. Eur Urol.

[16] Motzer RJ, Jonasch E, Agarwal N (2022). Kidney Cancer, Version 3.2022, NCCN Clinical Practice Guidelines in Oncology. J Natl Compr Canc Netw.

[17] Campbell MT, Bilen MA, Shah AY (2018). Cabozantinib for the treatment of patients with metastatic non-clear cell renal cell carcinoma: a retrospective analysis. Eur J Cancer.

[18] Chanzá NM, Xie W, Bilen MA (2019). Cabozantinib in advanced non-clear-cell renal cell carcinoma: a multicentre, retrospective, cohort study. Lancet Oncol.

[19] Jung KS, Lee SJ, Park SH (2018). Pazopanib for the treatment of non-clear cell renal cell carcinoma: a single-arm, open-label, multicenter, phase II study. Cancer Res Treat.

[20] McDermott DF, Lee JL, Ziobro M (2021). Open-label, single-arm, phase II study of pembrolizumab monotherapy as first-line therapy in patients with advanced non-clear cell renal cell carcinoma. J Clin Oncol.

[21] Koshkin VS, Barata PC, Zhang T (2018). Clinical activity of nivolumab in patients with non-clear cell renal cell carcinoma. J Immunother Cancer.

[22] Hutson TE, Michaelson MD, Kuzel TM (2021). A single-arm, multicenter, phase 2 study of lenvatinib plus everolimus in patients with advanced non-clear cell renal cell carcinoma. Eur Urol.

